# Bayesian network modeling of risk and prodromal markers of Parkinson’s disease

**DOI:** 10.1371/journal.pone.0280609

**Published:** 2023-02-24

**Authors:** Meemansa Sood, Ulrike Suenkel, Anna-Katharina von Thaler, Helena U. Zacharias, Kathrin Brockmann, Gerhard W. Eschweiler, Walter Maetzler, Daniela Berg, Holger Fröhlich, Sebastian Heinzel

**Affiliations:** 1 Department of Bioinformatics, Fraunhofer Institute for Algorithms and Scientific Computing (SCAI), Sankt Augustin, Germany; 2 Bonn-Aachen International Center for IT, University of Bonn, Bonn, Germany; 3 Department of Psychiatry and Psychotherapy, Tübingen University Hospital, Tübingen, Germany; 4 Department of Neurodegeneration, Hertie Institute for Clinical Brain Research, University of Tübingen, Tübingen, Germany; 5 Department of Internal Medicine I, University Medical Center Schleswig-Holstein, Campus Kiel, Kiel, Germany; 6 Institute of Clinical Molecular Biology, Kiel University and University Medical Center Schleswig-Holstein, Campus Kiel, Kiel, Germany; 7 German Center for Neurodegenerative Diseases, University of Tübingen, Tübingen, Germany; 8 Geriatric Center, Tübingen University Hospital, Tübingen, Germany; 9 Department of Neurology, Christian-Albrechts University, Kiel, Germany; 10 Institute of Medical Informatics and Statistics, Kiel University, University Hospital Schleswig-Holstein, Kiel, Germany; Policlinico Riuniti of Foggia: Neuroscience Department, S.C. Ospedaliera of Neurology-Stroke Unit, ITALY

## Abstract

Parkinson’s disease (PD) is characterized by a long prodromal phase with a multitude of markers indicating an increased PD risk prior to clinical diagnosis based on motor symptoms. Current PD prediction models do not consider interdependencies of single predictors, lack differentiation by subtypes of prodromal PD, and may be limited and potentially biased by confounding factors, unspecific assessment methods and restricted access to comprehensive marker data of prospective cohorts. We used prospective data of 18 established risk and prodromal markers of PD in 1178 healthy, PD-free individuals and 24 incident PD cases collected longitudinally in the Tübingen evaluation of Risk factors for Early detection of NeuroDegeneration (TREND) study at 4 visits over up to 10 years. We employed artificial intelligence (AI) to learn and quantify PD marker interdependencies via a Bayesian network (BN) with probabilistic confidence estimation using bootstrapping. The BN was employed to generate a synthetic cohort and individual marker profiles. Robust interdependencies were observed for BN edges from age to subthreshold parkinsonism and urinary dysfunction, sex to substantia nigra hyperechogenicity, depression, non-smoking and to constipation; depression to symptomatic hypotension and excessive daytime somnolence; solvent exposure to cognitive deficits and to physical inactivity; and non-smoking to physical inactivity. Conversion to PD was interdependent with prior subthreshold parkinsonism, sex and substantia nigra hyperechogenicity. Several additional interdependencies with lower probabilistic confidence were identified. Synthetic subjects generated via the BN based representation of the TREND study were realistic as assessed through multiple comparison approaches of real and synthetic data. Altogether our work demonstrates the potential of modern AI approaches (specifically BNs) both for modelling and understanding interdependencies between PD risk and prodromal markers, which are so far not accounted for in PD prediction models, as well as for generating realistic synthetic data.

## Introduction

Parkinson’s disease (PD) is characterized by progressive neurodegeneration that has usually advanced for many years before it is clinically diagnosed [1]. In addition to old age, a multitude of risk markers, such as genetic factors, lifestyle, environmental factors, (comorbid) diseases (e.g., diabetes) as well as biomarkers (e.g., low plasma urate levels, hyperechogenicity of the substantia nigra) have been shown to indicate an increased risk of PD in prospective studies [2, 3]. Moreover, prodromal markers like depression, autonomous dysfunction, REM-sleep behavior disorder (RBD), subtle motor signs and pathological dopaminergic imaging [3–5] may already indicate early neurodegenerative processes that can ultimately lead to the clinical diagnosis of PD. The International Parkinson and Movement Disorder Society (MDS) research criteria for prodromal PD [3, 6] have been designed to review and continually update the predictive values of risk and prodromal markers of PD. This is indicated by the positive and negative likelihood ratio (LR+, LR-) calculated from a 2x2 table of prospective data: marker present/absent and incident PD diagnosis/healthy. Moreover, these criteria proposed a naïve Bayesian classifier approach for the calculation of the probability that an individual is in the prodromal phase. With age providing an *a-priori* probability of prodromal PD as derived from epidemiological evidence [7], the individual profile of risk and prodromal markers, i.e. constellations of LR+ and LR- values, allows the calculation of an *a-posteriori* probability of prodromal PD [6]. While these criteria have repeatedly been shown to be highly specific, sensitivity may depend upon marker selection, depth of assessment and time to PD diagnosis and possibly specific subtypes of prodromal PD [8, 9]. While having the advantages of being both evidence-based as well as practical, several limitations and assumptions are inherent to this approach such as statistical independence assumption of risk markers and prodromal markers as well as age. These assumptions are most likely not fulfilled in reality and should be addressed to improve PD prediction accuracy. For example, many prodromal markers increase in prevalence with advancing age irrespective of a future PD diagnosis, which may decrease their specificity for PD prediction in an age-dependent manner [10]. Also, marker prevalence and their predictive value for PD may be sex-specific, e.g., as previously suggested for depression [10]. Thus, the predictive value for PD as currently assigned to the presence, absence or borderline status of a particular marker, may partially depend on age as well as constellations of the presence and absence of other markers in the profile of an individual. Marker co-occurrences may (partially) depend on e.g., methodological aspects of data collection and marker assessment, shared bio-pathological pathways and clinical comorbidity features. Such interdependencies can influence the actual predictive value of specific marker constellations.

The heterogeneity of PD in its clinical as well as in its prodromal phase may be partially explained by different subtypes of the disease [8, 9], e.g., subtypes differentiated by the site of initiation, risk and prodromal marker progression profiles of pathology (brain-first vs. body-first) [12, 13], and temporal dynamics in the prodrome of PD. However, as comprehensive data of (major) prospective population-based cohorts is often not jointly accessible, early (prodromal) subtyping, predictive values of markers (and their interdependencies) by subtype is still largely restricted to highly selected and specific clinical populations such as RBD patients. Consequently, an evidence-based understanding of prodromal PD to improve PD prediction and aid the (subtype-specific) recruitment of future early intervention trials in prodromal PD is challenged by the unavoidable statistical biases of each clinical study due to predefined patient selection criteria.

Artificial Intelligence (AI) approaches, such as Bayesian networks (BNs) [14], may offer possible solutions to these challenges, as 1) interdependencies of markers can be modelled, 2) BNs can be used to realistically simulate prospective cohorts, which could–at least partially–help to overcome restrictions posed by data privacy, and 3) access to such synthetic, comprehensive (population-based) cohort data. Thereby, both the consideration of more generalizable evidence underlying PD prediction as well a more differentiated investigation and understanding of prodromal PD subtypes may be supported and possibly help to inform the design and recruitment for early intervention trials in prodromal PD.

The present study has the aim to model a BN with the interdependencies between longitudinal data of risk and prodromal markers of PD and incident PD status of a large prospective cohort (TREND study)

## Materials and methods

### Overview of the TREND study data

The TREND study is a prospective cohort study which has been conceptualized for the investigation of markers that may help to predict PD and/or Alzheimer’s disease (AD). The cohort is partly population-based and partly enriched with individuals with an increased PD/AD risk by selectively recruiting participants based on the presence of olfactory loss, depression, and/or possible RBD. Comprehensive assessments of risk and prodromal markers of neurodegeneration, and e.g., neurological, neuropsychiatric and quantitative motor testing as well as biosampling in 1,201 individuals (aged 50+ years at baseline), have been performed every two years (baseline in 2009/2010, follow-up 1 to 4; follow-up 5 is currently ongoing). For more information, visit https://www.trend-studie.de/english. The study was approved by the local ethics committee (Medical Faculty, University of Tübingen; 444/2019BO2). All participants provided written informed consent. Study data were collected and managed using REDCap electronic data capture tools hosted at University of Tübingen [9].

Cohort participants in part had a delayed inclusion in the study (at follow-up 1) and some participants missed single waves or dropped out of the study (retention rate at follow-up 4: 72.4%). Therefore, the number of individual visits instead of the wave number of the TREND study is considered in the present work. For some participants the duration between two visits may occasionally be longer than two years. After excluding individuals with PD or parkinsonism at visit 1, we included data of 1178 (98.08%) participants collected at four consecutive visits (Tables 1 and 2) as the number of individuals with five visits was substantially lower (n = 545, 45.4%). Changes in sample size between visits as well as missingness of marker assessments per visit are shown in Tables 1 and 2. For the BN approach missingness both due to study drop-out (until visit 4) as well as due to missingness of marker assessment at single visits was considered for the imputation of data (see below and Supporting Information).

**Table 1:** Age and risk markers of PD in the prospective TREND study.

Features		Visit 1	Visit 2		Visit 3		Visit 4	
*Risk markers*	Category	PD-free (n = 1178)	PD-free (n = 1070)	Incident PD (n = 6)	PD-free (n = 981)	Incident PD (n = 5)	PD-free (n = 880)	Incident PD (n = 5)
*Age at visit 1*		63 (58, 68)	65 (60, 70)	74 (70, 74)	67 (62, 72)	75 (73, 76)	69 (64, 74)	70 (68, 71)
*Sex*	Male	599 (51%)	546 (51%)	5 (83%)	503 (51%)	5 (100%)	464 (53%)	3 (60%)
	Female	579 (49%)	524 (49%)	1 (17%)	478 (49%)	0 (0%)	416 (47%)	2 (40%)
*PD family history*	No	1010 (86%)	917 (86%)	3 (50%)	840 (86%)	4 (80%)	751 (85%)	4 (80%)
	Yes	168 (14%)	153 (14%)	3 (50%)	141 (14%)	1 (20%)	129 (15%)	1 (20%)
*Polygenic risk score*	Marker absent	247 (21%)	218 (20%)	2 (33%)	204 (21%)	1 (20%)	181 (21%)	3 (60%)
	Borderline	500 (42%)	459 (43%)	2 (33%)	420 (43%)	2 (40%)	380 (43%)	1 (20%)
	Marker present	252 (21%)	237 (22%)	1 (17%)	218 (22%)	1 (20%)	197 (22%)	0 (0%)
	Missing	179 (15%)	156 (15%)	1 (17%)	139 (14%)	1 (20%)	22 (14%)	1 (20%)
*GBA mutation*	No	1126 (96%)	1021 (95%)	6 (100%)	938 (96%)	2 (40%)	845 (96%)	4 (80%)
	Yes	52 (4%)	49 (5%)	0 (0%)	43 (4%)	3 (60%)	35 (4%)	1 (20%)
*SN hyperechogenicity*	SN-	839 (71%)	771 (72%)	1(17%)	714 (73%)	3 (60%)	641 (73%)	3 (60%)
	SN+	210 (18%)	191 (18%)	5 (83%)	178 (18%)	2 (40%)	168 (19%)	2 (40%)
	Missing	129 (11%)	108 (10%)	0 (0%)	89 (9%)	0 (0%)	71 (8%)	0 (0%)
*Occupational pesticide exposure*	No	878 (75%)	855 (80%)	1(17%)	839 (86%)	1 (20%)	813 (92%)	3 (60%)
	Yes	19 (2%)	18 (2%)	0 (0%)	17 (2%)	0 (0%)	17 (2%)	0 (0%)
	Missing	281 (24%)	197 (18%)	5 (83%)	125 (13%)	4 (80%)	50 (6%)	2 (40%)
*Occupational solvent exposure*	Yes	774 (66%)	753 (70%)	1 (17%)	739 (75%)	1 (20%)	714 (81%)	2 (40%)
	No	129 (11%)	125 (12%)	0 (0%)	122 (12%)	0 (0%)	117 (13%)	1 (20%)
	Missing	275 (23%)	192 (18%)	5 (83%)	120 (12%)	4 (80%)	49 (6%)	2 (40%)
*Diabetes type II*	No	1131 (96%)	1015 (95%)	5 (83%)	922 (94%)	5 (100%)	826 (94%)	4 (80%)
	Yes	47 (4%)	55 (5%)	1 (17%)	59 (6%)	0 (0%)	54 (6%)	1 (20%)
	Missing	0 (0%)	0 (0%)	0 (0%)	0 (0%)	0 (0%)	0 (0%)	0 (0%)
*Physical inactivity*	No	542 (46%)	343 (32%)	1 (17%)	754 (77%)	4 (80%)	701 (80%)	3 (60%)
	Yes	142 (12%)	98(9%)	0 (0%)	223 (23%)	1 (20%)	176 (20%)	2 (40%)
	Missing	494 (42%)	629 (59%)	5 (83%)	4 (0%)	0 (0%)	3 (0%)	0 (0%)
*Non-smoking*	Marker absent	535 (45%)	478 (45%)	2 (33%)	438 (45%)	3(60%)	396 (45%)	0 (0%)
	Borderline	533 (45%)	509 (48%)	4 (67%)	470 (48%)	2(40%)	422 (48%)	4 (80%)
	Marker present	109 (9%)	83 (8%)	0 (0%)	72 (7%)	0 (0%)	61 (7%)	1 (20%)
	Missing	1 (0.1%)	0 (0%)	0 (0%)	1 (0.1%)	0 (0%)	1 (0.1%)	0 (0%)

Summary statistics of age and risk markers of PD-free individuals and incident PD cases at different visits, absolute and relative (%) frequencies of marker presence or median (inter-quartile range in brackets) are given unless specified otherwise. Sample sizes per visit are indicated. Missingness of marker data within a given visit is indicated, i.e. not considering longitudinal study dropout. Percentage values indicate the relative frequency of marker presence/absence/borderline/missingness within PD-free and incident PD groups, respectively, and within each visit. GBA, glucocerebrosidase; PD, Parkinson’s disease; SN, substantia nigra.

**Table 2:** Prodromal markers of PD in the prospective TREND study.

*Features*		Visit 1	*Visit 2*		*Visit 3*		*Visit 4*	
*Prodromal markers*	Category	PD-free (n = 1178)	PD-free (n = 1170)	Incident PD (n = 6)	PD-free (n = 981)	Incident PD (n = 5)	PD-free (n = 880)	Incident PD (n = 5)
Hyposmia	Marker absent	913 (78%)	860 (80%)	1 (17%)	747 (76%)	0 (0%)	665 (76%)	1 (20%)
	Borderline	244 (21%)	186 (17%)	4 (67%)	194 (20%)	5 (100%)	164 (19%)	4 (80%)
	Marker present	17 (1%)	4 (0.4%)	0 (0%)	24 (2%)	0 (0%)	42 (5%)	0 (0%)
	Missing	4 (0.3%)	20 (2%)	1 (17%)	16 (2%)	0 (0%)	9 (1%)	0 (0%)
Constipation	Marker absent	1015 (86%)	891 (83%)	4 (67%)	810 (83%)	2 (40%)	758 (86%)	5 (100%)
	Borderline	139 (12%)	135 (13%)	2 (33%)	113 (12%)	2 (40%)	90 (10%)	0 (0%)
	Marker present	15 (1%)	23 (2%)	0 (0%)	28 (3%)	1 (20%)	28 (3%)	0 (0%)
	Missing	9 (1%)	21 (2%)	0 (0%)	16 (2%)	0 (0%)	4 (1%)	0 (0%)
Excessive Daytime Somnolence	No	0 (0%)	32 (3%)	1 (17%)	383 (39%)	1 (20%)	839 (95%)	5 (100%)
	Yes	1178 (100%)	1037 (97%)	0 (0%)	586 (60%)	0 (0%)	2 (0.2%)	0 (0%)
	Missing	0 (0%)	10 (<1%)	5 (83%)	12 (1%)	4 (80%)	39 (4%)	0 (0%)
Symptomatic Hypotension	Marker absent	918 (78%)	793 (74%)	5 (83%)	746 (76%)	5 (100%)	738 (84%)	3 (60%)
	Borderline	230 (20%)	218 (20%)	0 (0%)	200 (20%)	0 (0%)	96 (11%)	1 (20%)
	Marker present	28 (2%)	52 (5%)	1 (17%)	27 (3%)	0 (0%)	43 (5%)	1 (20%)
	Missing	2 (0.2%)	7 (1%)	0 (0%)	6 (0.6%)	0 (0%)	3 (0.3%)	0 (0%)
Urinary Dysfunction	Marker absent	733 (62%)	691 (65%)	4 (67%)	637 (65%)	2 (40%)	631 (72%)	3 (60%)
	Borderline	391 (33%)	286 (27%)	1 (17%)	269 (27%)	2 (40%)	192 (22%)	1 (20%)
	Marker present	51 (4%)	82 (8%)	1 (17%)	65 (7%)	1 (20%)	53 (6%)	1 (20%)
	Missing	3 (0.3%)	11 (1%)	0 (0%)	10 (1%)	0 (0%)	4 (1%)	0 (0%)
pRBD	No	1116 (95%)	1041(97%)	6 (100%)	959 (98%)	4 (80%)	852 (97%)	4 (80%)
	Yes	54 (5%)	28 (3%)	0 (0%)	22 (2%)	1 (20%)	28 (3%)	1 (20%)
	Missing	8 (1%)	1 (<0.1%)	0 (0%)	0 (0%)	0 (0%)	0 (0%)	0 (0%)
Subthreshold parkinsonism (MDS-UPDRS-III)	No motor deficit	1006 (85%)	980 (92%)	0 (0%)	913 (93%)	1 (20%)	784 (89%)	1 (20%)
	Borderline motor deficit	120 (10%)	66 (6%)	1 (17%)	39 (4%)	2 (40%)	63 (7%)	0 (0%)
	Subthreshold parkinsonism	52 (4%)	24 (2%)	5 (83%)	29 (3%)	2 (40%)	33 (4%)	4 (80%)
	Missing	0 (0%)	0 (0%)	0 (0%)	0 (0%)	0 (0%)	0 (0%)	0 (0%)
Depression	No	830 (70%)	735 (69%)	3 (50%)	666 (65%)	4 (80%)	599 (68%)	4 (80%)
	Yes	348 (30%)	335 (31%)	3 (50%)	315 (32%)	1 (20%)	281 (32%)	1 (20%)
	Missing	0 (0%)	0 (0%)	0 (0%)	0 (0%)	0 (0%)	0 (0%)	0 (0%)
Global cognitive deficits	No	971 (82%)	911 (85%)	6 (100%)	856 (87%)	4 (80%)	788 (90%)	4 (80%)
	Yes	194 (16%)	142 (13%)	0 (0%)	111 (11%)	1 (20%)	81 (9%)	1 (20)
	Missing	13 (1%)	17 (2%)	0 (0%)	14 (1%)	0 (0%)	11 (1%)	0 (0%)

Summary statistics of prodromal markers of PD-free individuals and incident PD cases at different time points, absolute and relative (%) frequencies of marker presence or median (inter-quartile range in brackets) are given unless specified otherwise. Sample sizes per visit are indicated. Missingness of marker data within a given visit is indicated, i.e. not considering longitudinal study dropout. Percentage values indicate the relative frequency of marker presence/absence/borderline/missingness within PD-free and incident PD groups, respectively, and within each visit. MDS-UPDRS-III, MDS-sponsored Unified Parkinson’s Disease Rating Scale, motor part 3; PD, Parkinson’s disease, pRBD, possible REM sleep behavior disorder.

While all of these participants were PD-free at the first visit, in total n = 24 incident PD cases were clinically diagnosed at follow-up based on UKBB and MDS diagnostic criteria [15]. The visit at which the conversion to PD occurred was considered. For 16 of the 24 incident PD cases diagnoses were made during Visits #2–4, the remaining 8 patients have been diagnosed with PD after Visit #4 at Visit #5 or #6. Unlike the status as incident PD case, the marker data of Visit #5 or #6 were not included in the BN as samples sizes were much smaller due to drop-out. Descriptive statistics of PD-free individuals and incident PD cases regarding demographic factors and risk and prodromal markers of PD are shown in Tables 1 and 2.

### Bayesian networks based approach

We propose a BN based approach [11] to model the interdependencies between different risk and prodromal markers of PD and their longitudinal changes in a multi-modal, multi-scale manner. BNs are probabilistic graphical models, where nodes represent variables and edges represent conditional probabilistic dependencies between them [12]. These conditional probabilistic dependencies are characterized by a conditional probability table (CPT) for each variable. These conditional distributions are specified by the network parameters [13]. The details about our BN modeling approach, including handling of missing values, are described in the Supporting Information.

In this work we compiled a BN of 10 risk markers and 8 prodromal markers as well as age. Risk and prodromal markers were selected based on the recent MDS research criteria for prodromal PD, and of which prospective data has been collected in the TREND study. The markers were assigned to different domains including: autonomic dysfunction (constipation, symptomatic orthostatic hypotension, erectile and urinary dysfunction), lifestyle features and related diseases (physical inactivity, non-smoking, diabetes type II), environmental features (occupational pesticide and solvent exposure), neuropsychiatric features (depression, global cognitive deficit), neurological features (subthreshold parkinsonism (based on MDS-UPDRS-III), possible REM-sleep behavior disorder (pRBD), hyposmia, substantia nigra (SN) hyperechogenicity), genetic factors (first-degree family history of PD, polygenic risk scores of PD, GBA mutations) and demographic factors (age, sex). Moreover, incident PD diagnosis (PD conversion based on neurological diagnosis during the time course of the study) was included as a node in the BN. Since erectile dysfunction was only assessed in males, this prodromal marker was not included in the final BN to avoid biases to the model. The details of marker assessment methods and definitions are provided in the Supporting Information. This also includes details regarding the handling of missing values.

We employed a BN to learn dependencies between these variables in a data-driven manner as a function of time. BNs result in a quantitative network representing statistical dependencies between variables [12, 14]. For each variable the probability to take a specific value, dependent on the values of its parents in the network, is inferred from the data. Notably, age (younger or older than 65 years) as well as risk and prodromal markers of PD have been discretized such that all variables indicate the presence or absence (or borderline status) of a marker in an individual TREND participant, as published previously for the TREND cohort [15, 16] and suggested by the MDS research criteria for prodromal PD [3, 6]. For each variable a CPT was estimated while learning the overall BN from data (See S2 Fig in S1 Appendix for CPT plots of each marker). Conversion to PD was defined as one node in the BN irrespective of the visit at which PD was diagnosed. Further details about the BN learning procedure including the constraints imposed and handling of missing values are reported in the Supplementary material.

**Fig 1:**
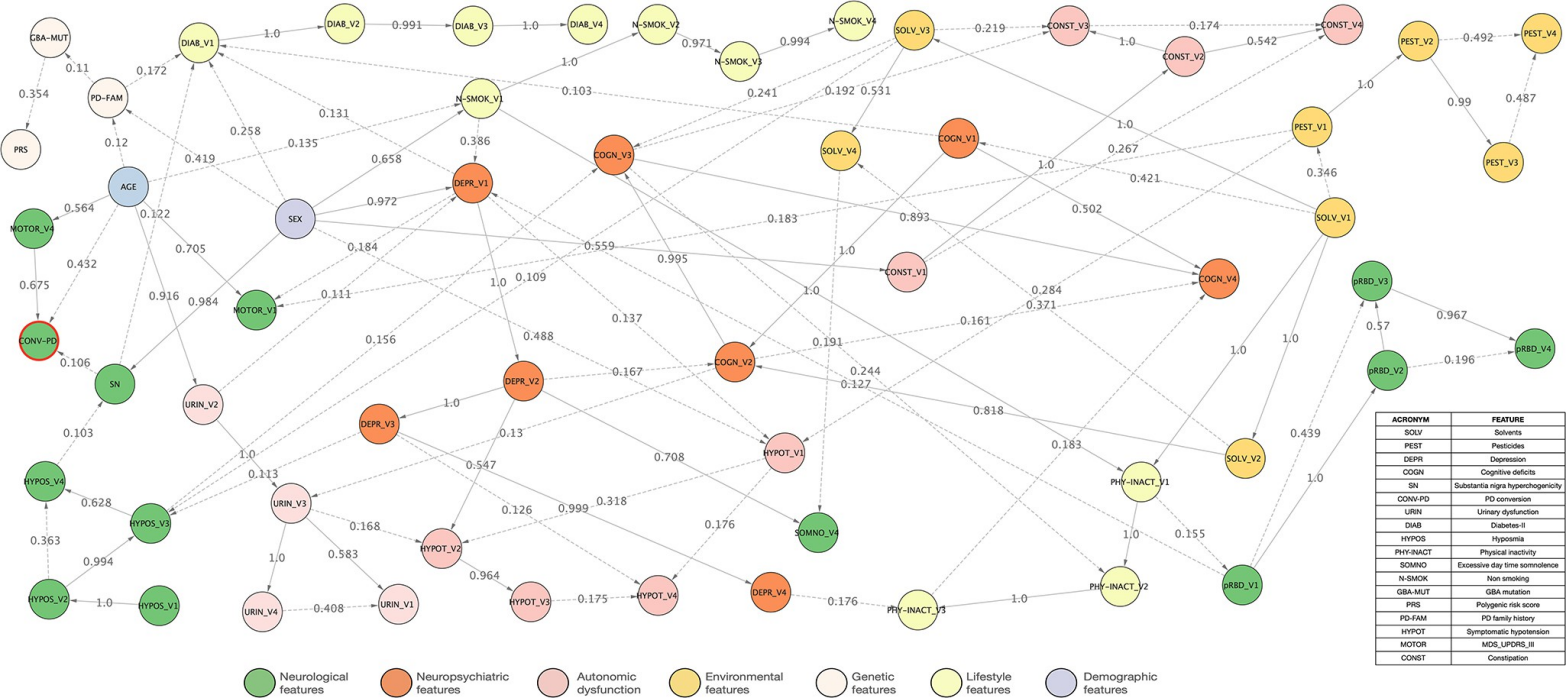
Interdependencies between different risk markers and prodromal markers of Parkinson’s Disease. The depicted Bayesian network represents interdependencies between variables learned from prospective TREND data. Domains of marker nodes are indicated by circle color. The node of the “Conversion to PD” is indicated by a red circle outline. Numbers on edges indicate the level of statistical confidence (bootstrap probability), and dashed edge lines indicate confidences <0.5 while solid lines indicate a confidence ≥0.5. A higher value indicates a higher confidence in the existence of a connection. Nodes isolated from the rest of the network are not shown. V indicates the respective visit number.

We trained a BN based on the data of all 1178 subjects using a non-parametric bootstrap [16] by randomly selecting n = 1178 for 1,000 times, with replacement, and for each of these 1,000 bootstrap samples we learned a complete BN structure. The relative frequency of observing a particular edge (i.e. conditional probabilistic dependency) among those 1,000 bootstraps was determined (see BN edges in Fig 1), and served as an indicator of the level of probabilistic confidence, i.e., a higher value means a stronger support by the data for the existence of the respective connection [16, 17]. A value of 1.0 indicates two specific nodes were interdependent in all the 1,000 learned BNs, a value of 0.5 indicates in 50% of the BNs an interdependency was observed. We selected a threshold of 0.5 as a conservative cutoff indicating high probabilistic confidence as only edges, which were found in a majority of bootstrap samples, should be interpreted.

### Evaluation via generating synthetic TREND subjects

BNs belong to the class of generative machine learning models. That means they learn the multivariate statistical distribution underlying the observed data in an unsupervised manner. Therefore, random samples drawn from the model correspond to synthetic subjects (see Supporting Information for details) [18, 19]. If those synthetic subjects are close to real ones, it can be assumed that the distribution learned by the BN represents well the original training data. Hence, we performed two different tests:

1. We generated the same number of synthetic individuals as real individuals for the data and then tested whether a conventional random forest (RF) classifier was able to separate between synthetic and real subjects within 10 times repeated 10-fold cross-validation scheme [5]. Therefore, we sequentially left out 1/10 of subjects and trained an RF on the remaining subjects to learn the discrimination between real and synthetic subjects. We used the left-out portion of the data to assess the prediction performance of the RF. We used the partial area under ROC curve (pAUC) at a pre-specified true positive rate of 99% for real subjects as a measure of the prediction performance. We chose the pAUC as adequate measure, because classification of all real subjects as real is aimed for while misclassification of synthetic ones as real does not constitute a negative classification property. The area under the ROC curve at which the detection rate for real subjects was between 99% and 100% served as an indicator of the validity of the synthetic TREND participants.
2. As a second test, we trained and evaluated the prediction performance of different machine learning models on real as well as synthetic data. More specifically, we here focused on the prodromal markers pRBD, hyposmia and depression. We trained a machine learning model (a random forest classifier) to test the ability of several variables to predict these prodromal markers at multiple visits. Outcomes at a subsequent visit were predicted by training the classifier on variables from the previous visit. For example, to predict the prodromal marker at visit 2, the classifier was trained on all the markers (measured longitudinally in the study) at visit 1. We either trained and tested the classifier on real subjects or trained the classifier on simulated / synthetic subjects generated by the BN and subsequently tested the classifier on real subjects. We evaluated the prediction performance of machine learning models using 10-fold cross validation repeated for 10 times. The overall dataset was randomly split into 10 folds, of which sequentially one of the folds was left out for testing the model, while the rest of the data was used for training. The prediction ability was measured via the area under the receiver operator characteristic curve (AUC) [20].

## Results

### Descriptive statistics

For each of the four visits, the descriptive statistics of the longitudinal data of risk markers (Table 1) and prodromal markers (Table 2) of PD-free individuals and incident PD cases is shown. Of 1178 subjects, 24 participants were clinically diagnosed with PD over the course of the prospective TREND (until visit 6).

### Bayesian network of risk and prodromal markers of PD in the TREND study

Fig 1 depicts the overall bootstrapped network structure of all connections learned from the TREND data. Estimated CPTs are presented in S2 Fig in S1 Appendix. A wide range in the level of confidence regarding the interconnectedness, i.e., statistical interdependence, was observed between several nodes and domain clusters of nodes. High probabilistic confidence of edges (>0.5, i.e. edges observed in the majority of BN bootstrap samples) between different markers in the BN was found for edges between age to subthreshold parkinsonism (MDS-UPDRS-III) and urinary dysfunction, sex to SN hyperechogenicity, depression, non-smoking and to constipation; depression to symptomatic hypotension and excessive daytime somnolence; solvent exposure to cognitive deficits and to physical inactivity; and non-smoking to physical inactivity. Pairwise co-occurrences of different markers showing edges with probabilistic certainties of >0.2 in the BN were shown and statistically tested for significance in Table 3. All of these edges also showed statistically significant co-occurrences between markers, except for sex and PD family history, sex and diabetes type-II (visit 1), occupational solvent exposure (visit 3) and constipation (visit 3), as well as GBA mutation carriers and PRS. These associations were no longer significant after accounting for multiple testing.

**Table 3:** Co-occurrence of markers.

Risk/prodromal marker A	Risk/prodromal marker B	Risk/prodromal marker B Participants (n)			p-value
		No	Borderline	Yes	
Male sex	Depression (V1)	471		128	<0.0001*
Female sex		359		220	
Male sex	Non-smoker (V1)	228	320	51	<0.0001*
Female sex		308	213	58	
Male sex	SN hyperechogenicity	453		146	<0.0001*
Female sex		515		64	
Male sex	Constipation (V1)	549	45	5	<0.0001*
Female sex		472	96	11	
Male sex	PD family history	529		70	0.013
Female sex		481		98	
Male sex	Symptomatic hypotension (V1)	508	83	8	<0.0001*
Female sex		412	147	20	
Male sex	Diabetes type II (V1)	567		32	0.024
Female sex		564		15	
Exposure to solvents (V1)	Cognitive deficits (V1)	302		86	<0.0001*
No exposure to solvents (V1)		678		112	
Exposure to solvents (V2)	Cognitive deficits (V2)	246		176	<0.0001*
No exposure to solvents (V2)		682		74	
Exposure to solvents (V3)	Cognitive deficits (V3)	201		237	<0.0001*
No exposure to solvents (V3)		668		72	
Exposure to solvents (V3)	Constipation (V3)	394	32	12	0.029
No exposure to solvents (V3)		626	88	26	
Exposure to pesticides (V1)	Symptomatic hypotension (V1)	13	20	1	<0.0001*
No exposure to pesticides (V1)		907	210	27	
Presence of depression (V2)	Day time somnolence (V4)	414		26	<0.0001*
Absence of depression (V2)		725		13	
Presence of depression (V2)	Symptomatic hypotension (V2)	213	200	27	<0.0001*
Absence of depression (V2)		588	122	28	
Non-smoker at visit (V1)	Physically active (V1)	107		429	<0.0001*
Borderline smoker (V1)		82		451	
Smoker (V1)		63		46	
Non-smoker (V1)	Depression (V1)	389		147	<0.0001*
Borderline smoker (V1)		382		151	
Smoker (V1)		59		50	
Presence of Global cognitive deficits (V2)	Physically active (V3)	224		85	<0.0001*
Absence of Global cognitive deficits (V2)		193		676	
GBA mutation carries	Polygenic risk score	21	24	7	0.002
GBA mutation non-carriers		226	655	245	
Age (> 65 years)	Conversion to PD	500		23	<0.0001*
Age (≤ 65 years)		654		1	
Age (> 65 years)	Subthreshold parkinsonism (V4)	462	31	30	<0.0001*
Age (≤ 65 years)		616	32	7	
Age (> 65 years)	Subthreshold parkinsonism (V1)	418	71	34	<0.0001*
Age (≤ 65 years)		588	49	18	
Age (> 65 years)	Urinary Dysfunction (V1)	269	220	34	<0.0001*
Age (≤ 65 years)		465	173	17	
Age (> 65 years)	Non-smoking (V1)	260	236	27	<0.0001*
Age (≤ 65 years)		276	297	82	

Statistical testing of the co-occurrence of risk and prodromal marker pairs (A & B) in the TREND data (including imputed data) as suggested by edges in the TREND BN of real data. P-values have been calculated based on a *χ*^2^-test and corrected for multiple testing using Holm’s method. Significant findings (after Holm-Bonferroni correction for multiple testing) are indicated by an asterisk. Findings remain significant in logistic regressions additionally accounting for age and sex. V, visit.

The BN revealed both expected as well as novel connections between risk and prodromal markers and the phenoconversion to PD. Plausibly, the nodes with edges directed to the conversion to PD comprised (prior) subthreshold parkinsonism indicated by MDS-UPDRS-III scores, age, and (with lower statistical confidence), SN hyperechogenicity. Further expected marker interdependencies were observed for edges pointing from depression and solvent exposure to global cognitive deficits, which itself was linked to physical inactivity while non-smoking was linked to physical inactivity. Edges pointing from depression to excessive daytime somnolence, pointing from solvent exposure and depression to hyposmia, or pointing from hyposmia to global cognitive deficits and to SN hyperechogenicity demonstrated further expected interdependencies.

Novel interdependencies were observed from non-smoking to depression; pesticide exposure to symptomatic hypotension; and edges with directionality from SN hyperechogenicity, global cognitive deficits, sex and PD family history to diabetes. Interestingly, constipation was dependent on sex, global cognitive and occupational solvent exposure. These dependencies were unexpected because they have not been reported in the established literature and/or in the context of (prodromal) PD. Surprisingly, little interdependencies were observed for pRBD, which was only linked to depression and received an edge from physical inactivity. This lack of interdependencies could be the consequence of assessing RBD by a questionnaire only (i.e. there was no polysomnography). Nodes with genetic features were not dependent on other markers except for sex being linked to PD family history, which itself was linked to diabetes.

Nodes of the same marker assessed at different timepoints were largely highly interdependent, except for subthreshold parkinsonism (MDS-UPDRS-III) for which visit 2 and visit 3, which were not linked to other nodes of the BN. MDS-UPDRS-III at visit 1 showed no edge with the corresponding nodes of other visits, but instead only received edges from depression and pesticide exposure at visit 1. An interactive Cytoscape network file of the BN is given in the Supporting Information.

### Evaluation via simulation of a synthetic TREND study cohort

**Fig 2:**
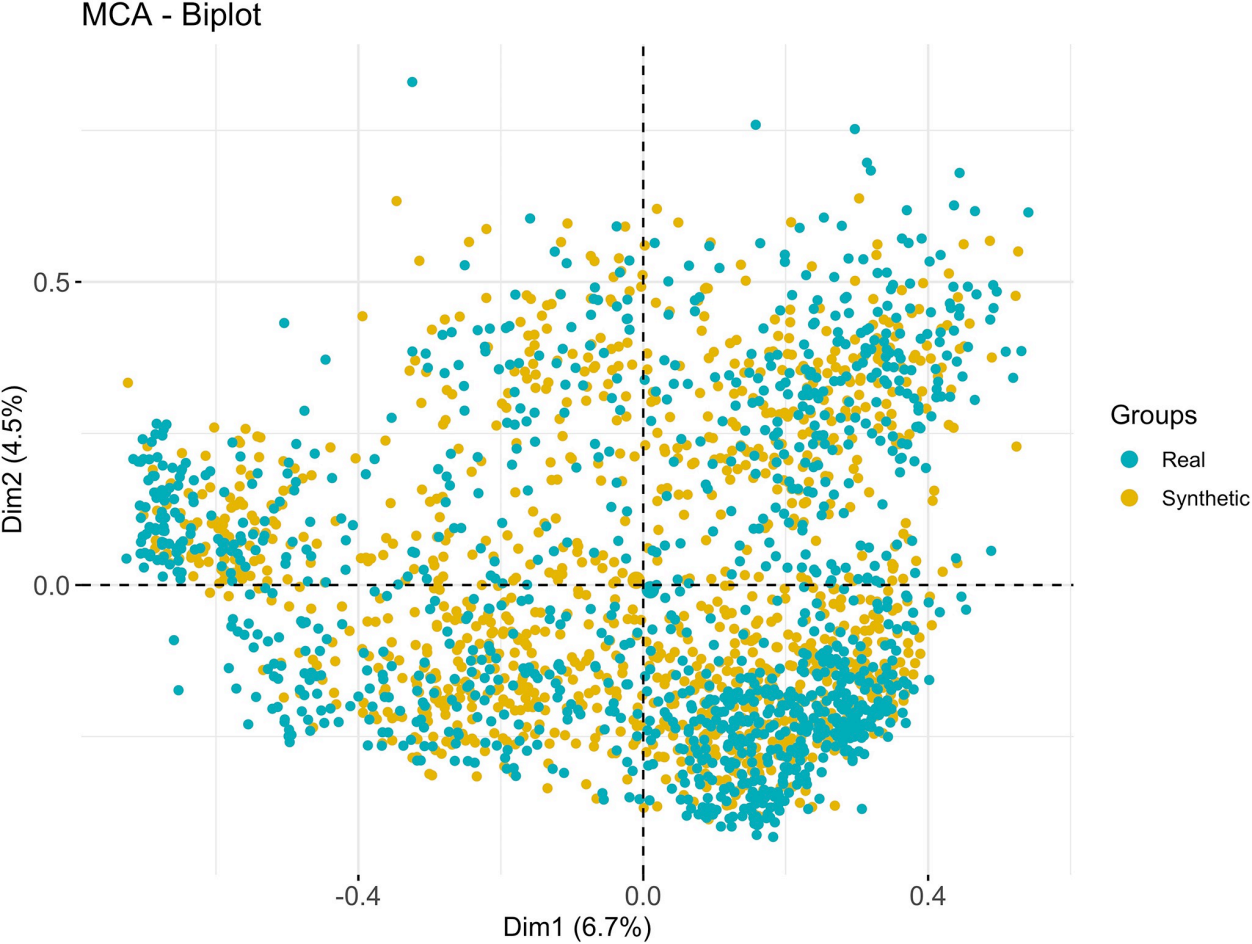
Multiple correspondence (MCA) analysis plot of prospective data of real (in blue) and simulated (in yellow) TREND participants.

The generative property of the BN allowed the simulation of synthetic versions of the prospective data of the TREND study and to extract individual synthetic participant profiles including age and the risk and prodromal markers of PD. Table 4 shows five arbitrary examples of synthetic subjects (from the synthetic cohort with the same sample size) and three real subjects together with their individual data (at visit 4) on age, sex, MDS-UPDRS-III, pRBD, depression, global cognitive deficits and PD conversion status. The Multiple Correspondence Analysis (MCA) [21] plot shown in Fig 2 indicates the similarity of synthetic subjects in relation to real ones. Further systematic comparisons of the distribution of individual variables and their correlation structure are presented in the (S2-S6 Figs in S1 Appendix). An RF classifier trained to discriminate between real and synthetic subjects only performed slightly better than chance level (pAUC 52%), indicating that both real and synthetic subjects cannot be reliably discriminated (S8 Fig in S1 Appendix).

**Table 4:** Real and synthetic marker profiles.

*Subjects*	*Age*	*Sex*	*MDS-UPDRS-III (V4)*	*pRBD (V4)*	*Depression (V4)*	*Global cognitive deficits (V4)*	*Conversion to PD*
*Synthetic subject # 1*	68	Male	No motor deficit	No	No	No	No
*Synthetic subject #2*	67	Female	No motor deficit	No	No	No	No
***Synthetic subject #3***	**68**	**Male**	**Subthreshold parkinsonism**	**No**	**No**	**Yes**	**Yes**
*Synthetic subject #4*	73	Female	No motor deficit	No	No	No	No
***Synthetic subject #5***	**69**	**Male**	**Borderline motor deficit**	**No**	**No**	**No**	**Yes**
*Real subject #1*	63	Female	No motor deficit	No	No	No	No
***Real subject #2***	**68**	**Male**	**Subthreshold parkinsonism**	**No**	**No**	**No**	**Yes**
***Real subject #3***	**70**	**Male**	**Borderline motor deficit**	**No**	**No**	**No**	**Yes**

Examples of synthetic and real subjects and their demographics, selected prodromal markers, subthreshold parkinsonism (MDS-UPDRS-III) and PD conversion status at visit 4. The rows in bold represent the similarity between the real and synthetic subjects’ data for incident PD cases. MDS-UPDRS-III, subthreshold parkinsonism indicated by the MDS-sponsored Unified Parkinson’s Disease Rating Scale; PD, Parkinson’s Disease, pRBD, possible REM-sleep behavior disorder. V, visit.

To further evaluate the synthetically generated TREND subjects we developed RF classifiers to predict for the individual participant, whether a participant would develop pRBD, hyposmia and/or depression at subsequent visits of the study. As outlined in the Methods part of this paper, corresponding classifiers were trained within a 10-times repeated 10-fold cross-validation, once on real subjects and once on synthetically generated subjects. To account for the possible variability due to the random sampling of synthetic subjects from the BN model, the process was repeated 10 times. Models were always tested on real patients.

Despite synthetic data generally showing a high similarity to real data, our results indicate a loss of ~10% AUC when training on synthetic compared to training on real subjects (S9 Fig in S1 Appendix). This could be due to slight differences between real and synthetic data regarding the distribution of individual variables (e.g. hyposmia, physical inactivity, see S5 Fig in S1 Appendix) as well as correlation structure (S7 Fig in S1 Appendix). Notably, RFs are a comparably complex machine learning method, which allows for modeling highly nonlinear structures.

Altogether these results highlight that synthetic data shared many patterns of real patient data and thus indicate a sufficient fit of the BN to the training data.

## Discussion

The present study shows the feasibility of learning and evaluating a BN based on prospective data of established risk and prodromal markers of PD in the TREND cohort of older PD-free individuals and incident PD cases. The BN model showed several expected as well as unexpected interdependencies between the markers, which may be explained by biological and clinical reasons for the co-occurrences of markers and/or by confounding due to practical and other methodological aspects of marker assessment. The multitude of marker interdependencies as revealed through the BN modelling could have important methodological implications for evidence-based PD prediction approaches as well as for the understanding of the interplay of different markers in the prodromal phase of PD.

The current established methodological approach of the MDS research criteria for prodromal PD [1, 2] uses a naïve Bayes classifier for the prediction of PD (or diagnosis of prodromal PD), which assumes that predictive values of risk and prodromal markers are statistically independent. However, based on our findings from BN model and pair-wise testing of co-occurrences of established PD markers in the prospective TREND cohort, we could show that for many of these predictive markers the assumption of statistical independence is most likely not met. Hence, concerns about the validity of the naïve Bayes classifier approach for PD prediction are raised.

While the number of incident PD cases was relatively low in the present study, robust and plausible interdependency was observed between MDS-UPDRS-III and the phenoconversion to PD. Also, age and SN hyperechogenicity were linked to the incidence of PD, which is expected as the prevalence of PD markedly increases with advancing age [1, 2] and SN hyperechogenicity is observed in 83% of PD patients [22]. However, for SN hyperechogenicity, a substantially lower probabilistic confidence was present in the bootstrapping of the BN models as may be partly explained both by low number of incident PD cases and by potential prodromal differences between distinct subtypes of the disease, e.g., the hypothesized brain-first vs. body-first prodromal PD subtypes [4, 6, 7].

Among risk and prodromal markers of PD, which have been shown to also play a role in other neurodegenerative and neuropsychiatric conditions, several interdependencies were observed in the BN model. In the following we only discuss those, which demonstrated a bootstrap confidence > 50%. Occupational solvent exposure has been associated with an increased risk of global cognitive impairment [23], which is consistent with their observed interdependency in the BN. As expected, current smokers were less physically active than former smokers and non-smokers explaining the edge between non-smoking and physical inactivity. Similarly, smokers were more frequently depressed than non-smokers. Given the known protective effects of smoking for PD [24] and increased PD risk due to physical inactivity and depression [25, 26], these often co-occurring factors may have opposing effects for individual PD risk estimates. Excessive daytime somnolence has been shown to be both a risk factor for depression as well as a frequent comorbid factor in depressed individuals, supporting their interdependency observed in the BN [27]. Diabetes received interesting edges from several nodes including SN hyperechogenicity, global cognitive deficits, sex and PD family history, and while their confidences were low, this finding might provide new hypotheses regarding biological prodromal mechanisms to be tested in future studies.

Several node interdependencies were unexpected and should be further investigated in independent cohorts. Possible RBD was only assessed using a self-report questionnaire, and while we applied the most specific criteria to determine the presence and absence of (possible) RBD [28], polysomnography would likely reveal a high false-positive rate among pRBD as a prevalence of polysomnography-proven RBD is less than 2% in the general, older population [29]. Low specificity of the assessment methods might have contributed to the lack of interdependencies between possible RBD and many other risk and prodromal markers of PD, including markers of autonomous dysfunction, which, together with RBD, may often co-occur in a body-first prodromal PD subtype [6].

Genetic risk markers of PD were, except for sex and diabetes, not interdependent with other risk and prodromal markers of PD, and while the number of GBA mutation carriers was low, a positive PD family history and a high polygenic risk score may increase the PD risk in a highly complex and multifaceted manner, which may partly explain the lack of their direct interdependency with other risk and prodromal markers.

While we expected age to be interdependent with several other markers frequent in old age (e.g., constipation, SN hyperechogenicity, hyposmia, global cognitive deficits), yet such edges were not observed in the BN and accounting for age did largely not alter effects of pair-wise co-occurrences in our analysis.

As expected, nodes of the same marker assessed at different visits were largely highly interdependent. MDS-UPDRS-III at visit 1 however showed no edge with the corresponding nodes of other visits, and the data of visits 2 and 3 were interdependent with one another, yet not connected to the BN. Possibly, motor deficits were either not (yet) apparent in some participants or motor deficits may have been confounded with non-PD related arthritic, tendon, bone or muscle complications at the first visit.

The present study has several limitations that need to be discussed. 1) Despite an excellent retention rate in the TREND study, participant attrition as well as missing data for single visits were observed in the longitudinal data. 2) Inter-visit dependency of markers, such as ratings of motor deficits, might in part be lowered due to changes of investigators between different waves of TREND data collection and assessment. 3) While a directionality of edges between markers is proposed by our BN approach, alternative directions between risk and prodromal markers as well as clinical features of (prodromal) PD may be observed in different models [30]. However, indeed directions not predefined by constraints were largely expected.

## Conclusion

In conclusion, the present study used a BN to disentangle the relationships of various established risk and prodromal markers in a large prospective cohort and showed that many of these markers are interdependent. Interdependencies of these predictive markers have not been accounted for in current PD prediction approaches, such as the MDS research criteria for prodromal PD [1, 2], hence raising concerns about their statistical validity. The BN of the TREND cohort contained data of a large sample of PD-free individuals, yet only a small sample of incident PD cases were available. Hence, an accurate PD prediction accounting for the interdependencies in marker profiles could not be derived from the given data. Overall, this work demonstrates the potential of modern AI approaches to advance our understanding of prodromal PD.

## Supporting information

S1 Appendix Definitions of risk and prodromal markers, Bayesian network specifications, methods of generating of synthetic data and additional data. (DOCX)

S1 File Interactive Cytoscape file of the Bayesian network. (ZIP)
